# Nitrofurantoin-induced splinter hemorrhages: A forgotten culprit

**DOI:** 10.1016/j.jdcr.2024.02.031

**Published:** 2024-03-08

**Authors:** Ana Sofia Acosta, Zainab Jafri, Krystina Khalil, Francisco Kerdel

**Affiliations:** a Florida Academic Dermatology Center, Coral Gables, Florida; b Larkin Community Hospital Palm Springs Campus, Hialeah, Florida; c Florida International University, Miami, Florida

**Keywords:** drug-induced nail changes, drug-induced splinter hemorrhages, nitrofurantoin, splinter hemorrhages

## Introduction

Splinter hemorrhages are formed by the extravasation of blood from the nail bed´s longitudinal vessels. These hemorrhages are more common in fingernails than toenails and present as black, brown, or purple in color. The blood attaches to the underlying nail plate and moves distally as the nail continues to grow. It is most commonly caused by trauma, systemic illnesses (ie, bacterial endocarditis or diabetes), cutaneous diseases (ie, psoriasis, onychomycosis, Darier disease, or Behcet), or drugs (ie, antibiotics, sedatives,^1^, ^2^ tyrosine kinase inhibitors) or epidermal growth factor receptor inhibitors.^3^ Nitrofurantoin, a lesser-known drug that may still be used in certain clinical settings, is a bactericidal antimicrobial that works through flavoprotein nitrofurantoin reductase to destabilize metabolites that disrupt ribosomal RNA, DNA, and intracellular components. It is active against most Gram-positive cocci and *Escherichia* *coli*, although *Pseudomonas* and *Proteus* are usually resistant to it and is generally used for lower urinary tract infections (UTIs). Herein, we present the case of an asymptomatic, 59-year-old Hispanic female who presented with splinter hemorrhages after a short course of nitrofurantoin for a UTI.

## Case report

**Fig 1:**
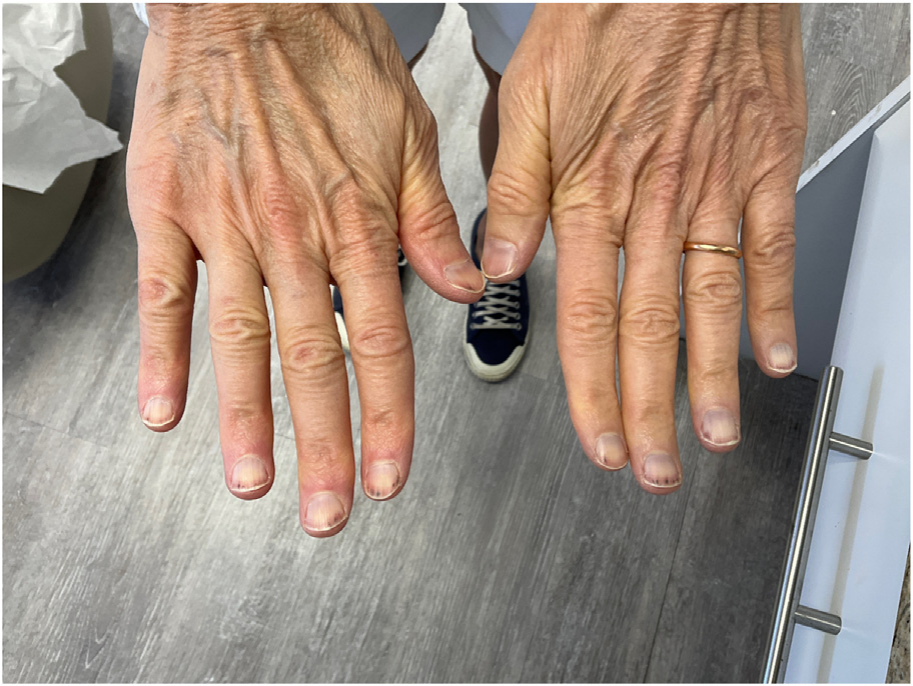
Thin *reddish-brown lines* or streaks located under the nail on distal nail plate of bilateral thumbs.

**Fig 2:**
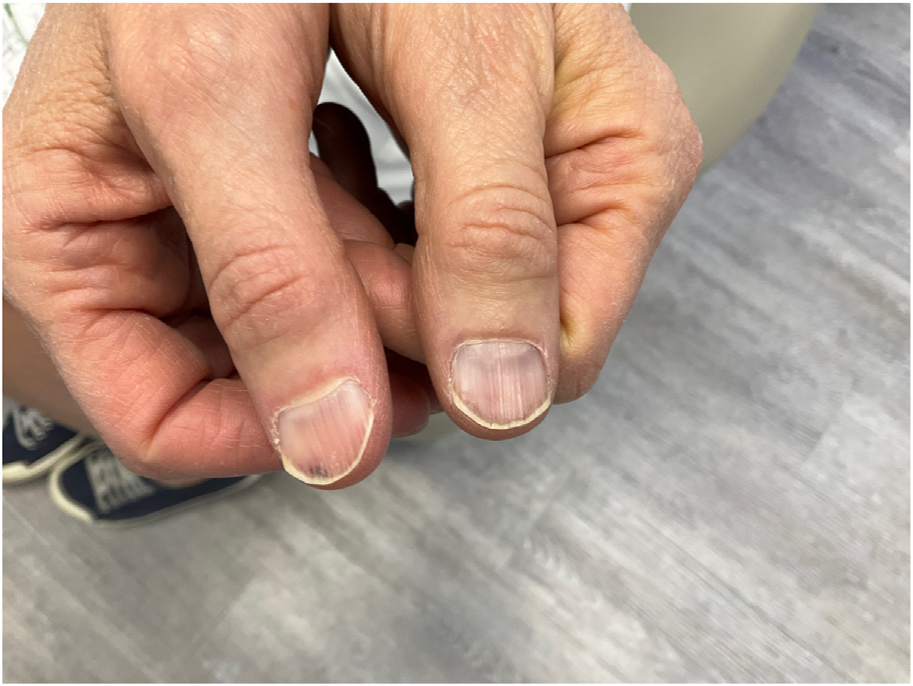
Thin *reddish-brown lines* or streaks located under the nail on distal nail plate of fingernails.

**Fig 3:**
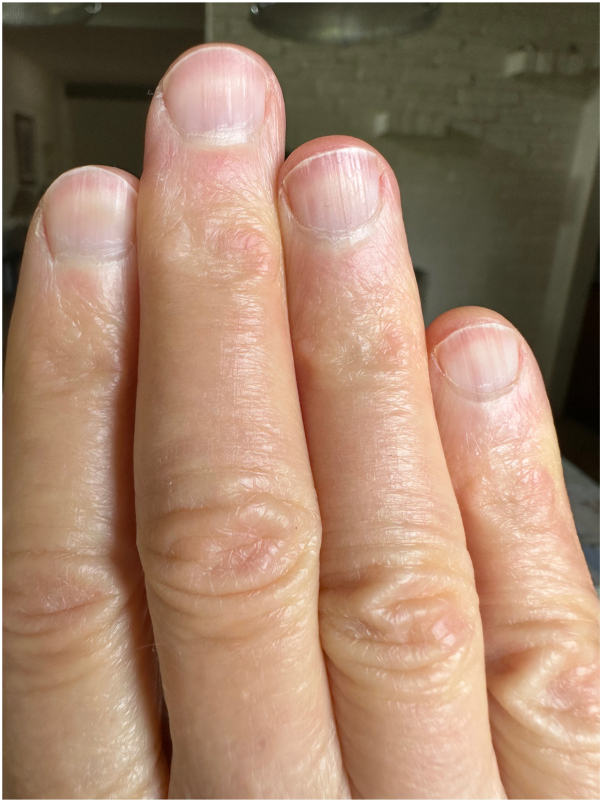
At 4 week follow-up, complete resolution of splinter hemorrhages upon discontinuation of drug, no apparent lesions on nail bed.

**Fig 4:**
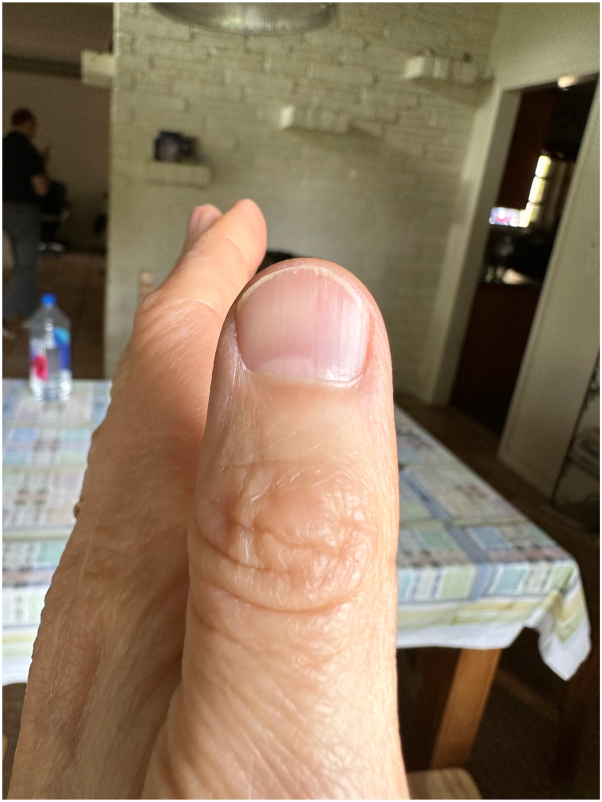
At 4 week follow-up, complete resolution of splinter hemorrhages upon discontinuation of drug, no apparent lesions on nail bed.

A 59-year-old Hispanic woman, Fitzpatrick II skin type, presented to the office with longitudinal splinter hemorrhages involving all 10 digits of the hands. Prior to her office visit, the patient presented to the emergency room due to her nail findings. She received a work up for bacterial endocarditis which consisted of 2 blood cultures, complete blood count, comprehensive metabolic panel, and chest x-ray; all of which were negative. At her office visit, the patient appeared healthy and asymptomatic on physical examination. Her only significant finding consisted of nonblanchable, distal, reddish-brown, linear hemorrhages without the presence of onycholysis (Figs 1 and 2) on all fingernails. Skin, hair, mucous membranes, and toenails were not affected. She denied a history of prosthetic heart valves, fever, chills, cardiac symptomatology, or recent physical activity involving trauma to the nails. She also denied recent use of medications such as nonsteroidal anti-inflammatory drugs, anticoagulants, and epidermal growth factor receptor inhibitors. Her past medical history included ductal hyperplasia treated with tamoxifen and a recent UTI treated with nitrofurantoin. Her symptoms began 2 days following initiation of nitrofurantoin. An extensive laboratory workup, including autoimmune lupus anticoagulant, anticardiolipin, beta glycoprotein, cytoplasmic anti-neutrophil antibody, perinuclear anti-neutrophil antibody, anti-proteinase 3, thromboembolic and infectious workup, and transthoracic echocardiogram, failed to show any evidence of bacterial endocarditis or other systemic disease. Only anti-myeloperoxidase antibodies were elevated (5.9 units, *n*: 0-0.9). The patient was advised to discontinue nitrofurantoin moving forward. At 4 week follow-up, the patient revealed no new lesions and markedly improved nail findings, with splinter hemorrhages fading (Figs 3 and 4).

## Discussion

The discovery of splinter hemorrhages dates back to the 1920s, when Blumer described subungual bleeding that looked identical to splinters under the nails.^4^ Excluding its common idiopathic nature, trauma and bacterial endocarditis as the most common etiology of this condition, multiple drugs and dermatologic conditions have been associated with splinter hemorrhages as well. This includes but is not limited to septic embolization, central venous and foley catheter infections, graft infections or failures, sepsis, meningococcemia, systemic lupus erythematosus, or vasculitis.^5^

Nitrofurantoin is an antimicrobial agent that has been used for many years in the setting of UTIs. Although it has been known to cause and/or associated with erythema nodosum, there have been limited reports of this drug causing splinter hemorrhages. This is likely due to less use of the drug by health providers due to the availability of more effective antibiotics. Despite the historic knowledge of oncohematologic drugs being a cause of splinter hemorrhages due to the antiangiogenic effects which impair the reparation of traumatized nail bed capillaries, we report a case of splinter hemorrhages affecting 10 fingernails after a week of nitrofurantoin, in the absence of cardiovascular symptoms and normal lab work.

A systematic review by Haber et al studied the possible etiologies and related conditions to splinter hemorrhages, and when drug-induced, splinter hemorrhages regressed after discontinuation of the offending drug. Nitrofurantoin has an inhibitory effect on adenisine diphosphate-induced platelet aggregation, which has been demonstrated in in vitro and in vivo studies. This has been supported by the prolongation of bleeding time and percent inhibition of aggregation being positively correlated with serum nitrofurantoin concentrations. Thus, the inhibitory mechanism on platelet aggregation poses as the culprit for the splinter hemorrhages found in our patient.^5^

Identifying splinter hemorrhages should raise concern for critical illness or infections and warrants a detailed clinical and laboratory examination. A careful medication history, even for short courses of antibiotics such as nitrofurantoin, could elucidate the cause of a rather daunting looking condition for the patient and prevent unnecessary testing in an asymptomatic patient. However, if no clinical and laboratory evidence of bacterial endocarditis or any other condition is found, splinter hemorrhages typically have no clinical significance.

Our case portrays the importance of obtaining a proper and thorough medical history, including risk factors, medication history, and family and surgical history to elucidate the cause of the condition. Additionally, when not associated with other symptoms in the absence of positive laboratory results and risk factors, it is important to advise patients that these symptoms should resolve without any intervention.

## Conflicts of interest

None disclosed.
